# The Improvement Effect and Mechanism of Longitudinal Ultrasonic Vibration on the Injection Molding Quality of a Polymeric Micro-Needle Array

**DOI:** 10.3390/polym11010151

**Published:** 2019-01-17

**Authors:** Shan Gao, Zhongjun Qiu, Junhao Ouyang

**Affiliations:** State Key Laboratory of Precision Measuring Technology & Instruments, Tianjin University, Tianjin 300072, China; gaoshan5@126.com (S.G.); ouyangjunhao@tju.edu.cn (J.O.)

**Keywords:** micro-injection molding, ultrasonic vibration, polymer material properties, polymer rheology, micro-needle array

## Abstract

A polymeric micro-needle array with high quality has been fabricated using a longitudinal ultrasonic-assisted micro-injection molding (LUμIM) method. To realize the practicability and stability in actual industrial processing, this paper is aimed at studying the improvement mechanism of ultrasonic vibration on the molding quality. The melt-filling process in the micro-needle array cavity is simulated, and the improvement effect of ultrasonic vibration is discussed. The enhancement effect of ultrasonic vibration on material properties of polypropylene and polymethylmethacrylate parts are experimentally investigated. The results show that in the manufacturing of the micro-needle array part using LUμIM, the mold-filling quality is improved by the enhanced melt filling capability and pressure compensation effect, which are caused by the increased corner viscosity gradient, reduced the filling time and melt viscosity under ultrasonic vibration. Material properties of both the semi-crystalline polymer and amorphous polymer could be enhanced by the transformation of micromorphology. It is proved that for a semi-crystalline polymer, this novel method could be employed as a material properties enhancement method, and an optimal excitation voltage of ultrasonic vibration is obtained to achieve the best material properties.

## 1. Introduction

Micro-needle array parts have already been applied in a number of domains, including medicine [1,2], biology [3,4], and micro-electromechanical systems [5,6], because of various excellent material properties, stable chemical properties, and good biological compatibility of polymer materials. Micro-injection molding (μIM) is an important method for the production of micro-needle array parts with advantages consisting of low manufacturing cost, short production cycle, mass-production, good repeatability, simple process, etc. [7,8]. However, due to the high aspect ratio of micro-needle array parts, the heat dissipation rate of the polymer melt in the microcavity is always very fast. As a result, the melt temperature will be rapidly decreased; subsequently, the melt viscosity will show a high rising gradient, and a frozen layer may sometimes be generated. The high melt viscosity and frozen layer in the microcavity will lead to molding defects and a low mold-filling quality, which often occurs in the production of microneedle array parts, and still cannot be solved perfectly by conventional injection molding technology [9,10]. These problems of molding quality limit the highly cost-efficient mass production of microneedle array parts with high integrity.

Generally, in the non-isothermal molding environment during μIM, the viscosity of the polymer melt could be decreased by increasing the mold temperature, melt temperature and injection speed, and the filling capability of melt and the replication quality of parts could be improved [11]. Some novel methods have been proposed to optimize μIM process parameters such as electric heating, steam heating, and infrared heating, which have been proposed for rapidly increasing the mold temperature [12]. However, the higher mold temperature, melt temperature, and injection speed would bring about more difficulties in the design and manufacture of the injection molding device; besides, these would also cause the waste of energy and decrease in molding quality [13,14]. To improve the molding quality of micropolymer parts without relying on a high standard of parameter adjustments, the ultrasonic vibration energy is brought into the micro-injection molding method.

Sato [15] developed the UμIM system for a precision injection molding process and carried out the molding experiment of optical lens. The experimental result showed that the weight of the lens was increased, the surface replication was improved, and the residual optical strain of the concave lens was decreased. Michaeli [16] put forward an ultrasonic plasticizing assisted micro-injection molding method to rapidly plasticize the polymer and improve the melt uniformity, subsequently to improve the mold filling quality and reduce manufacturing cycle. Masato [17] built an ultrasonic-assisted ejection system in micro-injection molding machine with the goal of reducing the ejection friction by decreasing the adhesion component of the frictional force, and the positive effect of ultrasonic vibration on the friction force during ejection was indicated based on experimental results. Hernández-Avila [18] employed an UμIM method for fabricating parts made of ultra-high molecular weight polyethylene and investigated the influence of the processing parameters on the structure, degradation, and mechanical properties by testing a miniaturized dog-bone shaped specimen.

During the UμIM, the ultrasonic vibration has not been directly applied to the polymer melt in the microcavity and the effects of ultrasonic vibration on the polymer melt, such as the improvement of the filling capability, were not yet played out well enough. Therefore, a longitudinal ultrasonic-assisted micro-injection molding (LUμIM) method was proposed and the improvement of mold filling quality has been clarified [19]. In this method, a longitudinal ultrasonic vibration directly acts on the melt through the core, which is integrated with an ultrasonic horn, and improves the filling quality of the mold with microstructures such as high aspect ratio structures or complex structures. The improvement effect of UμIM on height, uniformity, and shrinkage of the needles were verified using a molded experiment for a microneedle array [20]. However, for the microneedle array part and other micro polymer parts, the effect of ultrasonic vibration on the mold-filling quality improvement and material properties enhancement, as well as their mechanism, are not clear, thus the practicability and stability in actual industrial processing for solving problems that might be encountered cannot be achieved.

In this paper, the improvement effect and mechanism of ultrasonic vibration on the molding quality of microneedle array parts, including the improvement of mold filling quality and enhancement of material properties, via LUμIM method is investigated. The simulation of flow field in mold filling process of microneedle array parts is carried out, and the effect of ultrasonic vibration is discussed. An injection molding experiments using the self-developed novel system are carried out under different excitation voltages of ultrasonic vibration, and two kinds of polymer parts, whose materials are semi-crystalline polymer and amorphous polymer, are fabricated. By measuring the material properties and crystallinity of the parts, the effect and mechanism of ultrasonic vibration on material properties of the molded microneedle array parts in LUμIM is expounded.

## 2. Materials and Methods

### 2.1. LUμIM System

**Figure 1:**
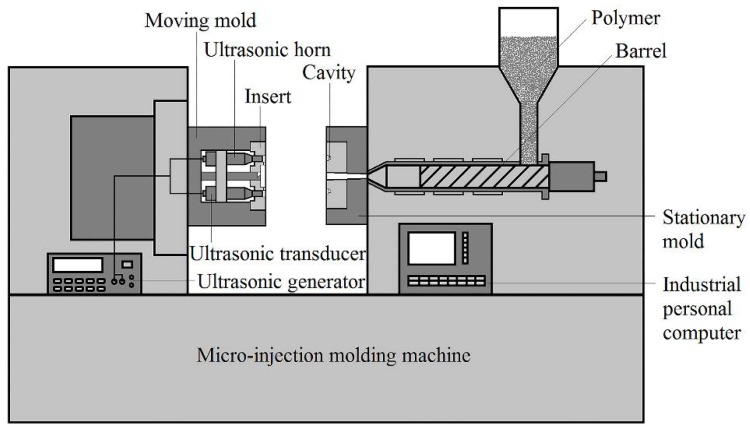
The schematic diagram of LUμIM system.

Figure 1 shows the schematic diagram of the LUμIM system, which consists of a precision injection molding machine and an ultrasonic vibration system. The ultrasonic vibration system includes ultrasonic transducers, which is mounted in movable half, ultrasonic horns, and an ultrasonic generator controlled by the precision injection molding machine FANUC ROBOSHOT S-2000i 50B (FANUC, Oshinomura, Japan). The ultrasonic vibration, whose propagation direction is parallel to the melt-filling direction in the microcavity, directly acts on the melt through the core, which is integrated with the ultrasonic horn for improving the filling quality of the mold. In order to make the effect of ultrasonic vibration more significant, the ultrasonic vibration is sustained throughout the whole molding process, especially including the injection stage, packing stage, and cooling stage. 

Generally, microneedle array parts could be made of both semi-crystalline and amorphous polymers. In order to analyze the effect of the mechanism of LUμIM, an experiment was carried out using a semi-crystalline polymer and an amorphous polymer as materials, where the former was used to investigate the material properties of the semi-crystalline polymer, especially the crystalline region, and the latter was used to clarify the material properties of the amorphous polymer and the amorphous region of semi-crystalline polymer. An X-ray diffractometer, Rigaku D/MAX-2500 (Rigaku, Akishima-shi, Japan), was employed to measure the crystallinity of the semi-crystalline polymer. A nano-indenter, Keysight Nano Indenter G200 (Keysight, Santa Rosa, CA, USA), was used to measure the hardness and elastic modulus of the semi-crystalline polymer part and amorphous polymer part for characterizing the material properties. Making more than five effective measurements for each experiment and ensuring that the standard deviation coefficient was less than 5%, and subsequently taking the average of the effective measurements as the measurement result.

### 2.2. Molding Materials

In this study, polypropylene (PP, JPC Novatec BC06N, JPC, Tokyo, Japan), which is a common semi-crystalline polymer with the advantages of high isotacticity and crystallinity, was employed in fabricating microparts using a LUμIM method to clarify the effect of ultrasonic vibration on material properties of the semi-crystalline polymer. The density of this PP material was 900 kg/m^3^ and the melt flow rate (MFR) was 60 g/10 min. Polymethylmethacrylate (PMMA, Mitsubishi Rayon VH001, Mitsubishi Rayon, Tokyo, Japan), which is a kind of commercially popular amorphous polymer in micro-injection molding, was selected to investigate effect of ultrasonic vibration on material properties of the polymer in amorphous region. The density of this PMMA material was 1190 kg/m^3^ and the MFR was 2.0 g/10 min.

### 2.3. Ultrasonic Vibration Parameters

Ultrasonic vibration parameters have great influences on the molding quality. In this paper, the frequency of the vibration was set to 55 kHz such that there was enough energy transmitted into the polymer. Meanwhile the cavitation effect, which may lead to molding defects, should be avoided. The ultrasonic vibration energy applied on the polymer melt could be controlled by adjusting the excitation voltage which can change the output energy of ultrasonic vibrator, the relationship between the excitation voltage *U* and the output energy of ultrasonic vibrator *E_out_* can be expressed as:

(1) $$E_{out}=\psi K_{r}^{2}W_{i}=\psi K_{r}^{2}U^{2}/Z$$

where *K_r_* is the electro-mechanical coupling factor of the piezoelectric ceramics; *W_i_* is the input electric energy; *ψ* is the energy attenuation coefficient of the ultrasonic horn, which depends on mechanical structures and material properties of the horn; and *Z* is the impedance of piezoelectric ceramics. The range of excitation voltage provided by the ultrasonic generator was from 0 V to 400 V to keep it below the breakdown voltage of the piezoelectric ceramics of ultrasonic transductor. The maximum theoretical displacement at the front of the ultrasonic horn was 28 μm. The duration of ultrasonic vibration was about 60 s in each molding cycle. 

## 3. Numerical Simulation

**Figure 2:**
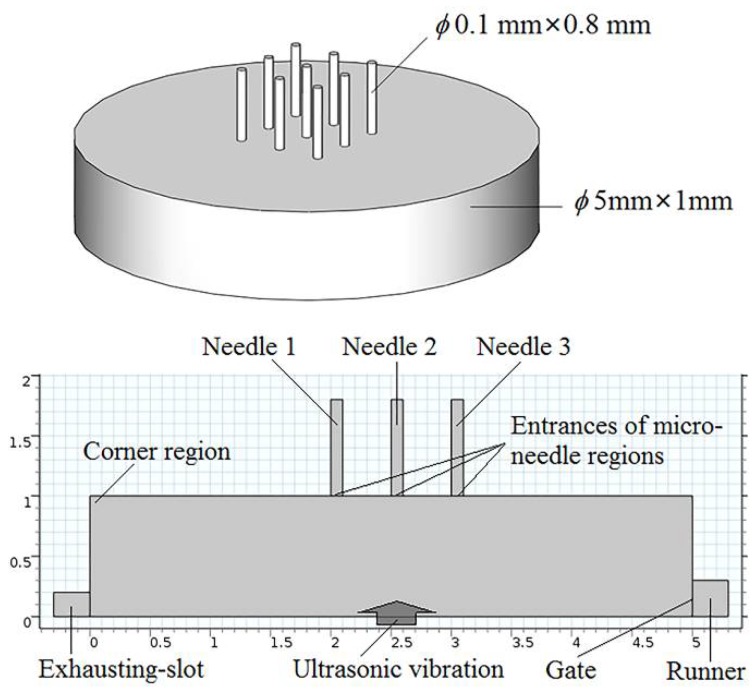
The model of the microneedle array part.

A numerical simulation of the melt flow field in the microneedle array cavity was carried out to clarify the specific effect mechanism and process of ultrasonic vibration on the mold filling quality. The model of the microneedle array part is as shown in Figure 2, in which each microneedle was 800 μm in height and 100 μm in diameter, and the volume of the part was approximately 19.68 mm^3^. Exhausting slots were set at the end of the cavity and the top of microneedles as in the actual processing. Based on flow simulation, the process of polymer melt flowing into the cavity was simulated. The theoretical fluid rheological models established in the previous work were taken in this simulation [21]. The melt flow velocity at the gate was set to be 6 m/s as the inlet boundary condition, and no additional pressure outlet boundary condition was set at the exhausting slot. PP was selected as an example, and other simulation parameters are consistent with the specific experimental parameters as shown in Table 1.

**Figure 3:**
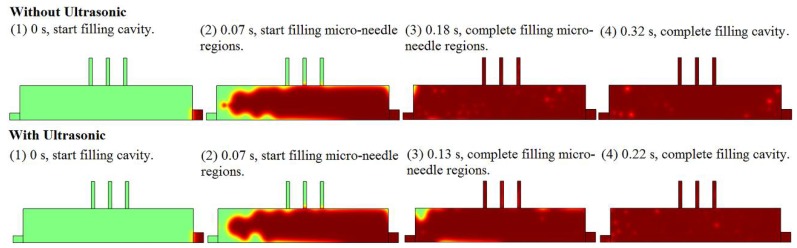
The filling process and volume fraction of melt in microneedle array cavity with and without the effect of ultrasonic vibration.

The filling process and volume fraction of melt in microneedle array cavity with and without the effect of ultrasonic vibration is shown in Figure 3 where the degree of red represents the volume fraction of the melt in the cavity. After the start of cavity filling, three time nodes are concerned, where they are the times the melt starts filling the microneedle regions, completes filling the microneedle regions, and completes filling the cavity. The criterion for filling completion is that there are no bubbles with a volume fraction of more than 50% in the region. It is shown that with the effect of ultrasonic vibration, the start times for filling the microneedle regions are the same, the time required to fill the microneedle regions can be reduced by 27.78% (from 0.18 s to 0.13 s), and the time required to fill the whole cavity can be reduced by 31.25% (from 0.32 s to 0.22 s). The shorter filling time can reduce the heat dissipation of the melt such that the melt can fill the cavity, especially the microneedle regions, with higher temperature and lower viscosity, which means achieving higher melt filling capability and less molding defects.

**Figure 4:**
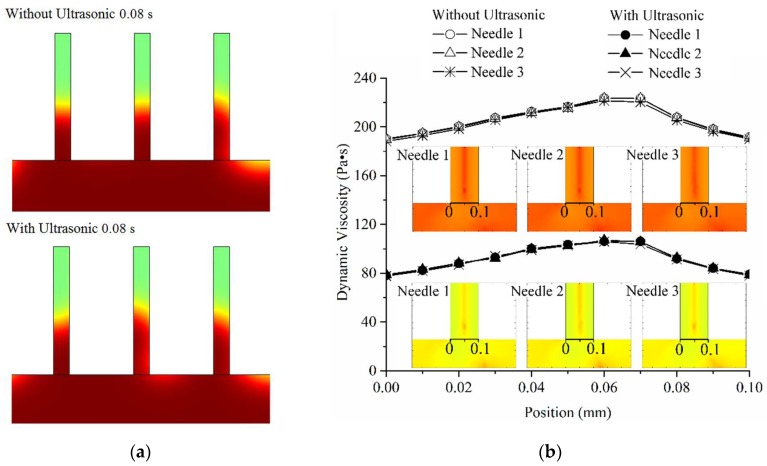
The melt filling and viscosity distribution of microneedle regions at 0.08 s with and without the effect of ultrasonic vibration: (**a**) the filling volume fraction of melt in micro-needle array cavity at 0.08 s with and without ultrasonic vibration, and (**b**) the melt viscosity distribution curves at the filling entrance transversal of microneedle regions with and without ultrasonic vibration.

The melt viscosity distribution at filling entrance transversal (black bold lines at entrances of microneedle regions in Figure 4b) of microneedle regions with and without the effect of ultrasonic vibration is shown in Figure 4. The selected observation time was 0.08 s when the filling of microneedle regions was under way. Obtained from Figure 4a, the filling flow field of the melt was similar between the two conditions of with and without ultrasonic vibration, and it shows that the filling order of the melt in the microneedle regions was not significantly changed by ultrasonic vibration. However, according to the melt viscosity distribution curves, the effect of ultrasonic vibration could decrease the melt viscosity value on the transversal by a large margin, although the distribution trends in the conditions of with and without ultrasonic vibration were mainly the same. Therefore, in the aspect of filling from large cavity to microregions, ultrasonic vibration improved the filling capability of the melt without changing the filling uniformity.

**Figure 5:**
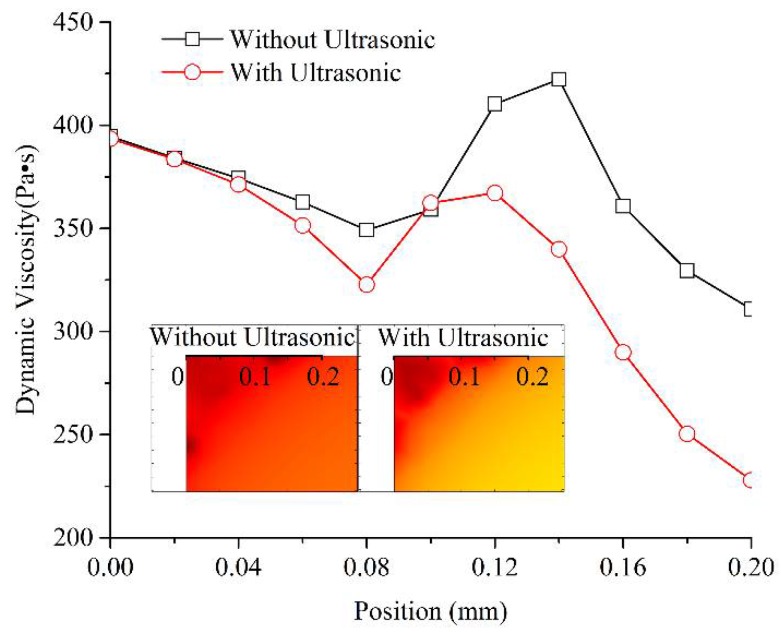
The melt viscosity distribution in the corner region with and without the effect of ultrasonic vibration.

During melt filling in the microcavity, the low shear rate and high melt viscosity often occurred in the corner region shown in Figure 2 and led to molding defects, such as a short shot and bubbles. The melt viscosity distribution in the corner region (on the black bold scale line) with and without the effect of ultrasonic vibration is shown in Figure 5. The position from 0 to 0.2 mm is the corner region where often generate high melt viscosity boundary layer and a short shot in micro-injection molding. The selected observation times were just the time to complete cavity filling with and without the effect of ultrasonic vibration, which were 0.22 s and 0.32 s, respectively. Under the effect of ultrasonic vibration, the melt viscosity in the corner region near the 0 position had no obvious change, but it was significantly decreased when the position was further away, from 0.1 mm to 0.2 mm and farther, and a high viscosity gradient was formed in the corner region. This means that when impacted by ultrasonic vibration, the melt could achieve a higher filling capability in the filling stage of the corner region. Then, in the holding stage, the low viscosity and high gradient were good for improving the pressure compensation effect. Thus, the final molded part could realize fewer molding defects and higher molding quality.

In summary, for the LUμIM method, ultrasonic vibration plays a positive role in improving the filling ability of polymer melt in the cavity of microneedle array part. First, by reducing the required filling times of microneedle regions and the whole cavity, the heat loss of the melt was reduced, the melt viscosity was reduced, and the filling capability and efficiency were improved. Second, the dynamic viscosity decreased greatly when the melt entering from the large-size cavity to the microneedle region, which improved the melt filling in the microcavity. Finally, the viscosity of the melt at the corner region was reduced and the gradient of the viscosity reduction was increased, and the filling capability of the melt in injection stage and the pressure compensation effect in the subsequent holding stage were improved, so as to reduce the molding defects and improve the molding quality. In the previous LUμIM experiments of microneedle array parts, under the effect of ultrasonic vibration, the average height of the microneedles increased from 520.58 μm to 581.25 μm, an increase of 11.65%. This increasing trend accorded with the filling improvement trend caused by the decrease of filling time and melt viscosity in the simulation results. The average shrinkage rate of the microneedles decreased from 0.89% to 0.78%, and decreased by 12.36%. This reducing effect accorded with the improvement of the pressure compensation effect caused by ultrasonic vibration in the simulation results [20].

## 4. Results and Discussion

**Figure 6:**
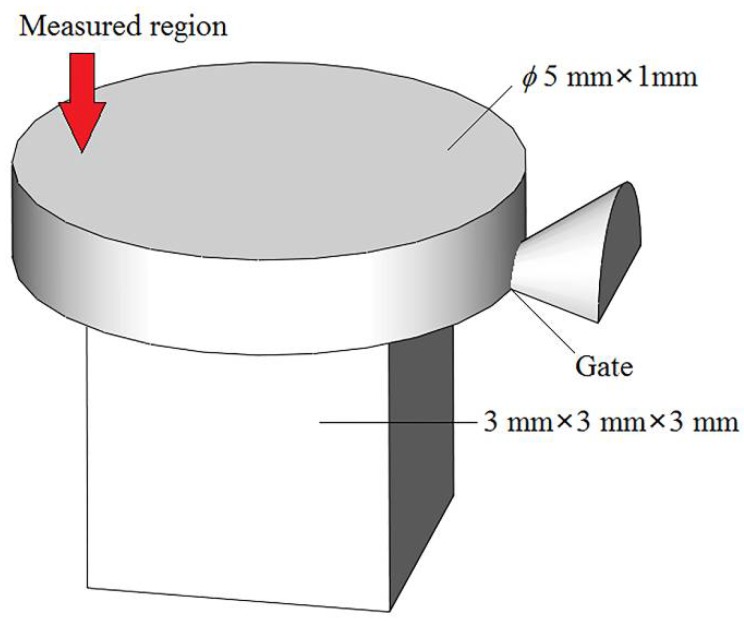
The diagram of the shape, dimensions, and the measured region of the base portion part.

The measured mechanical properties of molded microneedle array parts were affected by various factors such as material properties, dimensional errors, and molding defects. In order to clarify the influence of ultrasonic vibration on the material properties of microneedle array parts, it is necessary to eliminate other factors in the injection molding affecting the measurement data. Therefore, the measurement of the needle portion had a large deviation, and the measurement of base portion can better clarify the effect of ultrasonic vibration on the material properties of microneedle array parts. LUμIM experiments were carried out using PP and PMMA materials with different excitation voltages of ultrasonic vibration, where the processing parameters are shown in Table 2, and the diagram of the shape, dimensions and the measured region of the base portion part is shown in Figure 6. The measured region is located approximately 1.5 mm away from the center of upper surface in the opposite direction to the gate. This location aims to avoid the unstable shear flow and microstructural distortion caused by the entrance effect near the gate, and on the other hand, to avoid the negative influence of possible sink marks near the center of the upper surface. Making nine measurements for each experimental condition, meanwhile ensuring more than five effective measurement results whose standard deviation coefficient is less than 5%, and subsequently taking the average of the effective measurement results, gave the final measurement result.

**Figure 7:**
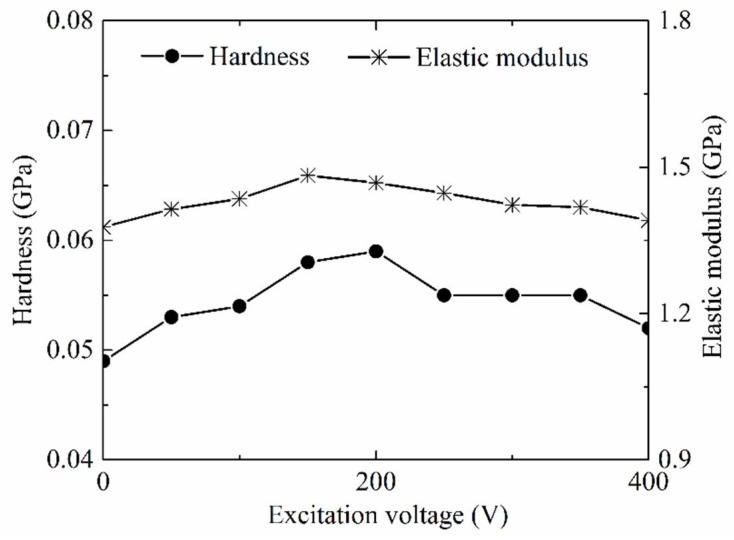
Effects of excitation voltage on the hardness and modulus of the PP parts.

As common material properties parameters, hardness and elastic modulus of the PP base portion part molded by LUμIM with the different excitation voltage of ultrasonic vibration were measured and used to evaluate the effect of ultrasonic vibration on the material properties of the semi-crystalline polymer, and the results are shown in Figure 7. Obviously, both of the hardness and elastic modulus of polymer part increased with the increase of the excitation voltage of ultrasonic vibration when the excitation was less than the optimal value. When the excitation voltage reached the optimal value, which was around 200 V in this research, the molded polymer part had the highest hardness and elastic modulus, and compared with the conventional micro injection molding, the hardness of the PP part fabricated using LUμIM increased by 20.4%, and the elastic modulus increased by 7.7%. When the excitation voltage exceeded the optimal value, the hardness and elastic modulus of the polymer decreased with the increase of excitation voltage. 

**Figure 8:**
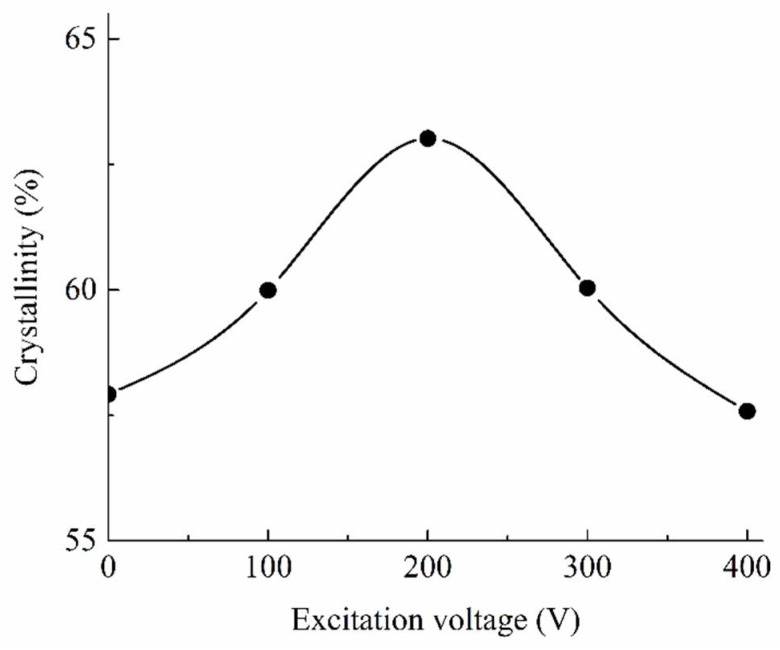
The crystallinity versus the excitation voltage of the PP parts.

The semi-crystalline polymer contained two regions, the crystalline region in which the arrangement of polymer chains is regular, and the amorphous region in which the arrangement of chains was disordered, meanwhile there was only an amorphous region in the amorphous polymer. Material properties of the semi-crystalline polymer were combined and determined by the morphology of these two regions [22]. In order to analyze the effect of transformation in crystalline region on the material properties of semi-crystalline polymer in LUμIM, the crystallinity of the molded PP base portion part was measured and the result is shown in Figure 8. As can be seen from the change of crystallinity, the ultrasonic vibration had an impact on the crystallization of PP, and in this research, when the excitation voltage was below 200 V, the crystallinity increased along with the increase of the excitation voltage; when the excitation voltage reached the optimal voltage 200 V, at which point, the semi-crystalline polymer had the highest crystallinity and the crystallinity in LUμIM was increased from 57.92% to 63.02% compared with the conventional micro-injection molding; then, the crystallinity decreased along with the increase of the excitation voltage when the excitation voltage exceeded the optimal value. This variation was caused by the effect of ultrasonic vibration on the crystal growth process and the formed crystal structure in LUμIM process.

During LUμIM process, the micromorphology in the crystalline region of the semi-crystalline polymer was recognized to be affected by ultrasonic vibration in many aspects. First, the high frequency reciprocating motion of polymer chains and the cavitation effect in the melt under the action of ultrasonic vibration led to high pressure, an intense heating effect, and a rapid heat transfer rate in local region of the micro cavity, especially near the upper surface of ultrasonic horn, and formed a uniform and compact arrangement of polymer chains, subsequently promoting the crystallization nucleation process and increasing the nucleation rate [23]. Second, the polymer chain tends to disorder under the effect of ultrasonic vibration, the common shish-kebab structure in traditional micro-injection molded part turned into a two-dimensional or three-dimensional crystal structure so that the space dimension of crystal growth was increased [24]. Third, the corrosion on the microcavity wall caused by cavitation damage and high frequency impact of melt under the effect of ultrasonic vibration promoted the nucleation type with the wall microstructure as the crystal nucleus [25]. Finally, during the propagation of ultrasonic vibration in the polymer melt, the internal friction and collision of polymer chains led to a heating effect on the melt, which could increase the nucleation rate [26]. Therefore, in summary, the crystallization rate of the semi-crystalline polymer was increased by ultrasonic vibration in LUμIM process. 

On contrary, the ultrasonic vibration also acted on the partially formed crystal region. The existing research results show that the ultrasonic vibration energy due to its impact of high-frequency vibration energy storage and cavitation effect in local area has the ability to reduce of the crystal size [27] and destroy the crystal structure [28], thus decrease the crystallinity. Therefore, in LUμIM, the effect of ultrasonic vibration on the crystallization of semi-crystalline polymer mainly includes two aspects, namely, the improvement of crystallization rate under ultrasonic vibration, which results in an increase in crystallinity of the molded polymer part, and the destruction of crystal structure under ultrasonic vibration, which results in a decrease in crystallinity of the molded polymer part. The crystallinity of the semi-crystalline polymer part molded by LUμIM is the comprehensive result of the above the specific experimental environment and process parameters.

According to the measurement result of crystallinity in Figure 8, when the excitation voltage of ultrasonic vibration did not reach the optimal value, the destruction of ultrasonic vibration on the crystal structure was not significant yet, and the improvement of ultrasonic vibration on the crystallization rate played a major role and the crystallinity increased along with the increase of the ultrasonic vibration energy. When the excitation voltage of ultrasonic vibration exceeded the optimal value, the destruction of ultrasonic vibration on the crystal structure played a more significant role than the improvement of ultrasonic vibration on the crystallization rate, and thus the crystallinity decreased along with the increase of the ultrasonic vibration energy.

**Figure 9:**
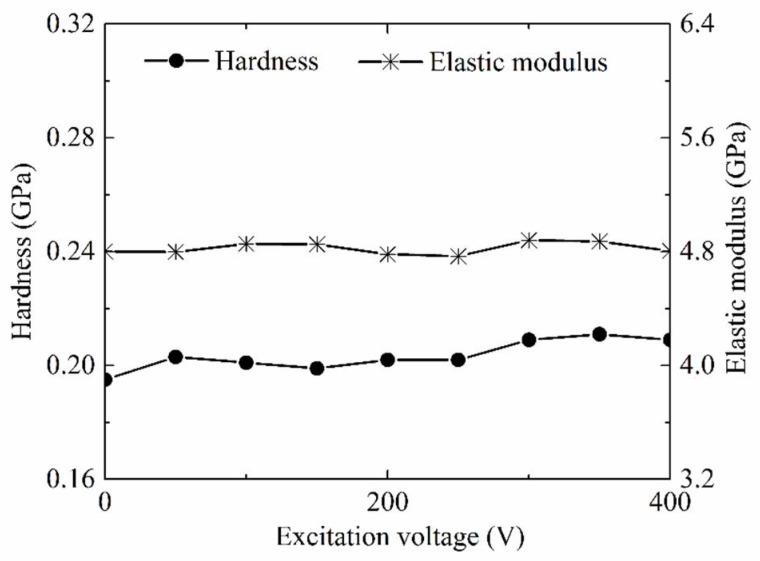
Effects of excitation voltage on the hardness and modulus of the PMMA parts.

It could be seen from the comparison Figure 7 and Figure 8 that, under ultrasonic vibration, the impact trends of the excitation voltage of ultrasonic vibration on the material properties and the crystallinity were basically the same, and the flex points all appeared in around a 200 V excitation voltage of the ultrasonic vibration. This consistency shows the effect of the microstructure transformation in the crystalline region caused by ultrasonic vibration on the material properties. In general, the material properties of the semi-crystalline polymer were affected by the micromorphology of both the internal crystalline region and the amorphous region. In order to investigate the effect of ultrasonic vibration on material properties of the internal amorphous region of semi-crystalline polymer, as well as the effect on material properties of amorphous polymer, which is only formed by amorphous region, the material properties of parts made of PMMA, as the sample amorphous polymer, molded by LUμIM with the different excitation voltage of ultrasonic vibration, were measured and are shown in Figure 9.

It could be observed from the measured result that the hardness increased slightly, and the elastic modulus had no significant change along with the increasing excitation voltage of ultrasonic vibration, and there was no obvious optimal voltage value. Compared with the hardness and elastic modulus in the conventional micro-injection molding, the highest hardness increased by 8.2% and the corresponding excitation voltage was 350 V, the highest modulus increased by 1.6%, and the corresponding excitation voltage was 300 V. Results show small changes under the effect of ultrasonic vibration, and the elastic modulus was even covered by data scattering as the value was less than the standard deviation coefficient which was 1.69% when the voltage was 300 V. These small changes of the measured material properties of the amorphous polymer showed that a tiny transformation of micromorphology in the amorphous region under ultrasonic vibration occurred and could affect the material properties of semi-crystalline polymer and amorphous polymer but not significantly in the LUμIM process. 

Accordingly, based on comprehensive consideration of the measured material properties of semi-crystalline polymer and amorphous polymer in fabrication of the microneedle array using LUμIM, it could be obtained that, for semi-crystalline polymer, the hardness and elastic modulus showed a trend of increasing first and then decreasing, where the highest crystallinity was generated with an optimal excitation voltage value. This trend was mainly caused by the transformation of micromorphology in the crystalline region and also a slight contribution given by that in the amorphous region. Meanwhile, for the amorphous polymer, the hardness and elastic modulus increased slightly with the increase of excitation voltage, which was due to the tiny change of micromorphology in amorphous region caused by ultrasonic vibration. For future research, the applicability of this material properties enhancement effect should be proved in the manufacturing of other micropolymer parts made of different kinds of materials, and based on theoretical analysis of aggregation behavior, where the proposed LUμIM method will hopefully be further developed as a surface properties enhancement method for micropolymer parts.

## 5. Conclusions

This study focused on the improvement effect and mechanism of ultrasonic vibration on the molding quality of microneedle array parts fabricated by LUμIM method, including the improvement of mold filling quality and enhancement of material properties. 

(1) The improvement effect on mold filling quality was clarified to be achieved by the melt filling capability enhancement. During melt filling in the cavity with the effect of ultrasonic vibration, the start time for filling microneedle region was not changed, the time required to fill the microneedle region could be reduced from 0.18 s to 0.13 s (reduced by 27.78%); furthermore, the time required to fill the whole cavity could be reduced from 0.32 s to 0.22 s (reduced by 31.25%). The filling order of the melt in the microneedle regions was not changed significantly with the added ultrasonic vibration. However, according to the melt viscosity distribution curves, the effect of ultrasonic vibration could decrease the melt viscosity value on the transversal region by a large margin, although the distribution trends in the conditions of with and without ultrasonic vibration were mainly the same. In the corner region that often generated a high melt viscosity boundary layer and a short shot in micro-injection molding, under the effect of ultrasonic vibration, the melt viscosity at the corner had no obvious change, but it showed a sharp decrease with a great gradient of dynamic viscosity when the position was away from the corner point. It was defined that the mold filling quality could be improved by the melt filling capability enhancement in the injection stage and the pressure compensation effect improvement in the holding stage due to the decreased required filling times to fill both the microneedle regions and the whole cavity, the reduced melt dynamic viscosity in the microcavity, and the increased viscosity gradient at corner under the effect of ultrasonic vibration. 

(2) The enhancement effect on material properties of the semi-crystalline polymer was realized, and an optimal excitation voltage of ultrasonic vibration to achieve the most significant enhancement effect was verified to exist. With that optimal excitation voltage, the hardness of the PP parts fabricated using LUμIM increased by 20.4%, and the elastic modulus increased by 7.7%. According to the experimental analysis of the crystallinity in the crystalline region and properties of amorphous region, this enhancement effect was considered to be caused by the transformation of micromorphology in both the crystalline region and amorphous region, and the former transformation plays a more significant role than the latter one. It could be confirmed that the proposed LUμIM method is an effective method to enhance material properties of the semi-crystalline microneedle array part and other micropolymer parts.

## Figures and Tables

**Table 1:** Parameters in the numerical simulation.

Parameters	Value
**Ultrasonic parameters**	
Amplitude (μm)	28
Frequency (kHz)	55
Duration (s)	60
**Molding process parameters**	
Injection speed (mm/s)	20
Melt temperature (°C)	230
Mold temperature (°C)	50

**Table 2:** Processing parameters in LUμIM of PP and PMMA base portion parts.

Material	Mold Temperature (°C)	Melt Temperature (°C)	Injection Speed (mm/s)	Packing Pressure (MPa)	Packing Time (s)	Cooling Time (s)
PP	50	230	30	30	20	40
PMMA	60	250	30	50	20	40
